# Impact of Personal Health Records and Wearables on Health Outcomes and Patient Response: Three-Arm Randomized Controlled Trial

**DOI:** 10.2196/12070

**Published:** 2019-01-04

**Authors:** Jeong-Whun Kim, Borim Ryu, Seoyoon Cho, Eunyoung Heo, Yoojung Kim, Joongseek Lee, Se Young Jung, Sooyoung Yoo

**Affiliations:** 1 Department of Otorhinolaryngology Seoul National University Bundang Hospital Seongnam Republic of Korea; 2 Office of eHealth Research and Businesses Seoul National University Bundang Hospital Seongnam Republic of Korea; 3 Graduate School of Convergence Science and Technology Seoul National University Suwon Republic of Korea; 4 Department of Family Medicine Seoul National University Bundang Hospital Seongnam Republic of Korea

**Keywords:** personal health record, lifestyle, sleep apnea, obstructive, delivery of health care, electronic health record, mobile health

## Abstract

**Background:**

Although using the technologies for a variety of chronic health conditions such as personal health record (PHR) is reported to be acceptable and useful, there is a lack of evidence on the associations between the use of the technologies and the change of health outcome and patients’ response to a digital health app.

**Objective:**

This study aimed to examine the impact of the use of PHR and wearables on health outcome improvement and sustained use of the health app that can be associated with patient engagement.

**Methods:**

We developed an Android-based mobile phone app and used a wristband-type activity tracker (Samsung Charm) to collect data on health-related daily activities from individual patients. Dietary record, daily step counts, sleep log, subjective stress amount, blood pressure, and weight values were recorded. We conducted a prospective randomized clinical trial across 4 weeks on those diagnosed with obstructive sleep apnea (OSA) who had visited the outpatient clinic of Seoul National University Bundang Hospital. The trial randomly assigned 60 patients to 3 subgroups including 2 intervention groups: (1) mobile app and wearable device users (n=20), (2) mobile app–only users (n=20), and (3) controls (n=20). The primary outcome measure was weight change. Body weights before and after the trial were recorded and analyzed during clinic visits. Changes in OSA–related respiratory parameters such as respiratory disturbance, apnea-hypopnea, and oxygenation desaturation indexes and snoring comprised the secondary outcome and were analyzed for each participant.

**Results:**

We collected the individual data for each group during the trial, specifically anthropometric measurement and laboratory test results for health outcomes, and the app usage logs for patient response were collected and analyzed. The body weight showed a significant reduction in the 2 intervention groups after intervention, and the mobile app–only group showed more weight loss compared with the controls (*P*=.01). There were no significant changes in sleep-related health outcomes. From a patient response point of view, the average daily step counts (8165 steps) from the app plus wearable group were significantly higher than those (6034 steps) from the app-only group because they collected step count data from different devices (*P*=.02). The average rate of data collection was not different in physical activity (*P*=.99), food intake (*P*=.98), sleep (*P*=.95), stress (*P*=.70), and weight (*P*=.90) in the app plus wearable and app-only groups, respectively.

**Conclusions:**

We tried to integrate PHR data that allow clinicians and patients to share lifelog data with the clinical workflow to support lifestyle interventions. Our results suggest that a PHR–based intervention may be successful in losing body weight and improvement in lifestyle behavior.

**Trial Registration:**

ClinicalTrials.gov NCT03200223; https://clinicaltrials.gov/ct2/show/NCT03200223 (Archived by WebCite at http://www.webcitation.org/74baZmnCX).

## Introduction

Lifelog data or patient-generated health data, considered important for precision medicine initiative implementation, form the next frontier in patient engagement and customized health care [1,2]. Through the availability of numerous devices and compatible mobile apps, patients can collect their own health-related lifestyle data, which can be aggregated with their clinical data into their own personal health record (PHR) [3-6]. A lifelog is a detailed chronicle of a person’s life involving large amounts of data. In recent years, the data are usually captured automatically by wearable technology or mobile devices. People who keep lifelogs about themselves are known as lifeloggers [7]. The purpose of lifelogging is to help users collect data for self-monitoring and reflection [8]. Technology has nearly reached the stage when all information, interesting or otherwise, generated in a lifetime by a single person can be assembled and queried relatively efficiently, creating a need for personal information management [9]. Lifelogging can be passive—one stores the by-products of the life one would have lived anyway—or active—one surrounds oneself with sensors and information capture tools to create as rich a picture of one’s life as possible [10].

Clinical wearables can be defined as health technology that can be worn by the patient. The wearables contain sensors and use a wireless connection to pass data to a smartphone or similar devices. Wearables are being used in the health care industry to help health care practitioners collect, analyze, and leverage patient data for clinical trials while also significantly improving patient care and overall quality of life [11]. Recent technology advancements in health care have the potential to close the communication and information gap between patients and providers. This has created a mandate for more interactive, demand-responsive mobile health (mHealth) tools that empower consumers to actively manage their own health [12]. Currently, there is an increasing awareness of the health care system’s responsibility to provide easily accessible ways for patients to be engaged in their own care by creating effective partnerships that lead to the patient’s ability to make competent and well-informed decisions [13]. Although an electronic PHR tethered to an electronic health record (EHR), also known as a patient portal, is currently recognized as a promising mechanism to support greater patient engagement, questions remain about how health care leaders, policy makers, and designers can encourage adoption by both providers and patients and what factors might contribute to sustained utilization [14]. In addition to user-specific characteristics (eg, age, sex, and diagnosis), studies should be conducted on the modifiable factors that affect use duration, to facilitate activities that promote continued use [15].

In our previous study [16], we showed that patients with chronic diseases are more likely to use a PHR system that is integrated into a comprehensive EHR. We also showed that patients with more chronic diseases tend to use PHR more actively, employing the self-administration function. Furthermore, our other previous clinical trial study [17] primarily aimed to demonstrate the development of an EHR-tethered PHR system in which a comprehensive EHR system that can retrieve data from a wearable device has been operated successfully for over 12 years as well as the efficacy of such a system paired with a lifelog data–driven intervention modality [18].

Our clinical trial focused on patients with obstructive sleep apnea (OSA), which is caused by complete or partial obstruction of the upper airway because OSA is closely related to individual lifestyle [19]. Obesity is the most important cause of OSA, and smoking and alcohol consumption are also important causes of OSA. Therefore, a better lifestyle can be a cornerstone of treatment for both obesity and OSA. The aim of the clinical trial was to help OSA patients to correct obesity through a healthier lifestyle and to obtain a better quality of sleep. Smartphone platforms (mHealth systems) are being considered as an innovative solution, thanks to the integration of the essential sensors to obtain clinically relevant parameters in the same device or in combination with wireless wearable devices [20,21]. A recent study from Cardiogram and the University of California, San Francisco, suggested that the Apple Watch can be used to test for OSA. This study indicates that wearable devices could provide an accessible, low-cost approach to evaluate OSA [22]. From the perspective of weight loss, there have been several studies trying to verify the effects of technology-based interventions. There were studies comparing in-person behavioral weight loss intervention with a technology-based system over a 3-month period in overweight adults [23,24]. A recent study tried a long-term observation with wearables during a 24-month study period [25]. The study stated that effective long-term treatments are needed to address the obesity epidemic and that numerous wearable technologies are unclear if these are effective in improving weight loss.

Our first clinical study was a preliminary study aimed at observing the weight loss impact for obesity patients of conventional care versus EHR-integrated PHR–based care after system development [17,23]. In particular, as a continuous development from previous studies, we aimed to reveal the effectiveness of a PHR–based health care app with 3 arms: mobile app and wearables, mobile app alone, and control. Furthermore, we examined the actual mobile lifelogs for each lifestyle categories: activity step counts, meals, sleep logs, and stress.

The primary objective of this more sophisticated 3-arm clinical trial design was to (1) explore the effect of a wearable device and mobile PHR app based on the patient’s weight loss and sleep-related health outcome for a research reproducibility perspective, (2) observe patient response as a proxy measure for patient engagement in EHR-integrated PHR use, and (3) study health app usability based on patients’ responses to automated lifestyle comments.

## Methods

### Design of Patient-Friendly Mobile Personal Health Record App

We designed a new mobile PHR app, *MyHealthKeeper* (Samsung Electronics Co, Seoul, South Korea), to be compatible with health data collection platforms of private companies and to be linked to our hospital EHR system. The user interfaces (Multimedia Appendix 1) were designed to be more patient-friendly when patients collect health-related lifestyle data on their own. A more sophisticated *MyHealthKeeper* PHR app to collect health-related lifestyle data was developed and tested in 2 different experimental patient groups (app and wearable user vs app-only user). The lifestyle data we tried to collect included weight, step counts as physical activity, food intake, sleep hours, and subjective strength of daily stress. The *MyHealthKeeper* app is largely composed of (1) logging according to the nature of the information and (2) screens registered in an accordion format, which can be used to navigate through the tabs. The app is composed of several subpages for recording daily meal, sleep log, stress, blood pressure, weight value, and synced activity step counts. When the user clicks the OK tab in the recording step, the objectives and guides, records, and daily span are presented, and the recording and modification functions are provided when each tab is clicked. The verification phase provides clinical advice that gives confirmation through weekly assessments. This clinician feedback comment is implemented on each page to improve communication between doctors and patients. The patient’s response to automated comment logs as well as other usage logs was collected and analyzed for each group.

In particular, we designed our app to be compatible with the Samsung Health platform, which is one of the biggest worldwide mobile platforms, and to be linked to our EHR-tethered PHR system. This is the first approach to be aligned with a private company platform and a tertiary general university hospital PHR in Korea. Therefore, we could develop flexible data collection techniques that can leverage user lifestyle logs in both devices and smartphones from users (walking steps in this study). Samsung Health (originally S Health) is a free app developed by Samsung that tracks various aspects of daily life contributing to well-being such as physical activity, diet, and sleep [24]. The app was installed by default only on some smartphones of the brand. It could also be downloaded from the Samsung Galaxy Apps store. The app is obviously compatible with Samsung fitness trackers and smartwatches on Samsung phones. Users get health data from their preferred tools, reducing the burden of collection, and Samsung Charm (Samsung Electronics Co, Seoul, South Korea), which was developed by Samsung Electronics and was used to collect daily activity data in this study.

### Design of a Clinical Trial

**Figure 1:**
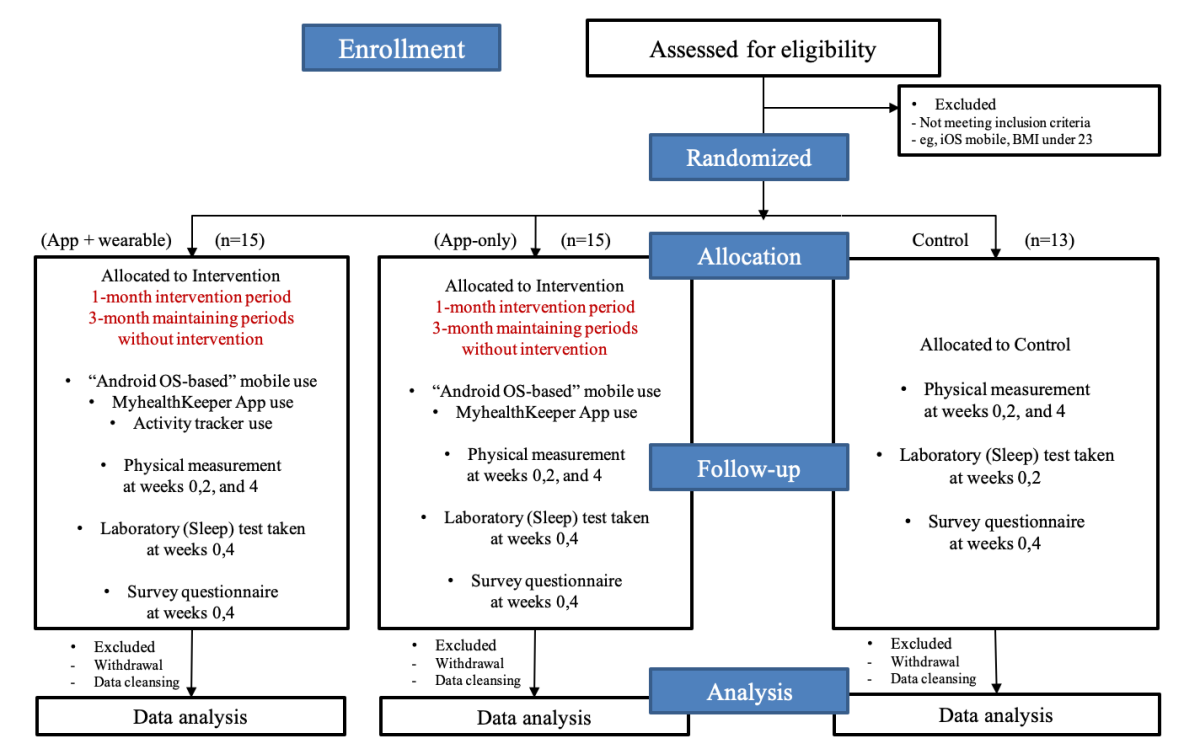
Clinical trial study design. BMI: body mass index; OS: operating system.

We conducted a prospective randomized clinical trial. Patients who visited the outpatient clinic of Seoul National University Bundang Hospital (SNUBH) were recruited from July 1 to August 31, 2017. We set the following inclusion criteria for enrollment in the trial: (1) diagnosis with OSA; (2) no cardiopulmonary disease, cancer, or other acute diseases; (3) body mass index (BMI) over 23 kg/m^2^; and (4) provision of prior consent to comply with self-management. We excluded patients who would not be able to use a mobile app and a wearable device and those who were pregnant. Figure 1 demonstrates the overall clinical trial design in this study. The participants had to follow the instructions of the app and wearable for 4 weeks, but they could use the app and wearable after 4 weeks on their own.

The participants were randomized into 3 groups: (1) app plus wearable group, (2) app-only group, and (3) control group. The app plus wearable group used the PHR app and Samsung Charm wearable activity tracker band. The step counts of the app-only group were collected from the mobile phone itself and delivered to the Samsung Health platform. The app plus wearable and app-only groups were instructed to use the apps as per the guideline. On the contrary, the control group was managed ad libitum. The control group did not receive the PHR app or the wearable device, and they did not use any other intervention than the verbal advice to lose weight during their visit to the clinician. All the participants in the 3 groups underwent sleep WatchPAT tests twice at weeks 0 and 4 to measure an objective respiratory parameter (apnea-hypopnea index per hour) during sleep. WatchPAT is a Food and Drugs Administration–approved portable diagnostic device that uniquely uses finger-based physiology and innovative technology to enable simple and accurate OSA testing while avoiding the complexity and discomfort associated with traditional airflow-based systems [25]. Body weight and height were also recorded twice at weeks 0 and 4.

This study was approved by the SNUBH Institutional Review Board (B-1504-296-302) and registered at the United States National Institutes of Health clinical trial registry (ClinicalTrails.gov registration number: NCT03200223).

### Personal Health Record–Based Patient Lifelog Data Collection

All study participants completed a paper-based survey at the first and last day of the trial. Subjective respiratory parameters during sleep in the survey questions included snoring frequency, snoring intensity, sleep apnea witnessed frequency, and daytime sleepiness. Snoring frequency, sleep apnea witnessed frequency, and daytime sleepiness were asked to answer by days in a week in the questionnaire (eg, How many days of sleep apnea a week is found by you or your family?). Snoring intensity, sleep apnea severity, and daytime sleepiness severity were asked to answer by 0- to 10-point scale in the questionnaire (eg, How loud is the snoring sound?; degree from 0 to 10). Furthermore, objective WatchPAT test was performed to examine deep and light sleep amount (percentage), Peripheral arterial tonometry Rapid Eye Movement (pREM; percentage), sleep latency (minutes), sleep efficiency, and number of wakes.

Meanwhile, the *MyHealthKeeper* app is designed and developed to collect manually recorded lifelogs and apply them to individual health management. Lifelog data collected by the app are categorized as weight, stress, meal, and sleep logs. Weight value, subjective stress, sleep time, and sleep satisfaction logs should be recorded once a day, whereas meal logs are recorded 3 times a day (breakfast, lunch, and dinner). Each meal record includes the input time and the amount on a 5-point scale. Physical activity is concerned with daily step counts, which are easily synced and delivered from the Samsung Health platform. The control group did not receive the lifestyle modification app and the wearable device. They received conventional care pertaining to lifestyle modification for achieving weight loss goals during the 4-week study period.

With *MyhealthKeeper* app, we calculated the average value of collected data from each lifelog such as physical activity, food intake, sleep time, daily stress, and weight. We simply combined all data logging scores for each patient. For example, if a user recorded every lifelog in a day, total score of the day is 5. Therefore, we could derive a star-shaped pentagon plot with 5 types of lifestyle log data. The mean area value for each plot is a range of 0 to 1 by every user. On the basis of this area value score, patients could be divided into 2 subgroups according to the median score. We defined a compliant subgroup with a score over 0.4 and noncompliant subgroup with a score under 0.4.

### Clinical Study Outcome Measure

The primary outcome of the trial was collection of lifestyle data and weight change. Any decrease in body weight during the study period (4 weeks) was defined as successful weight reduction. The secondary outcomes were changes in the subjective and objective respiratory parameters during sleep.

Body weights before and after PHR–based clinical intervention were recorded and analyzed. The BMI of each participant is defined as the body mass divided by the square of the body height and is expressed in units of kg/m^2^; the difference between BMI before and after the study was analyzed at the end of the study period. Any decrease in body weight during the study period (4 weeks) was defined as successful weight reduction. It is very important that the measurement be taken using the same method and in the same conditions to ensure uniformity between participants and in the same participant over time. In our study, a skilled nurse helped to measure the patient’s body weight in the hospital health checkup center using the conventional health checkup process (place and dress).

### Statistical Analysis Method

Results are presented as means (SD). Differences in various parameters between the PHR–based intervention group and the control group were analyzed using the chi-square test as appropriate. Paired *t* test was used to examine changes in primary or secondary outcomes in the groups. All statistical analyses were performed using R version 3.0.2 developed by R Core Team (R Foundation for Statistical Computing, Vienna 2013), and a *P* value of <.05 was considered statistically significant.

## Results

### Architecture of New MyhealthKeeper Linked to Samsung Health and Hospital Electronic Health Record

**Figure 2:**
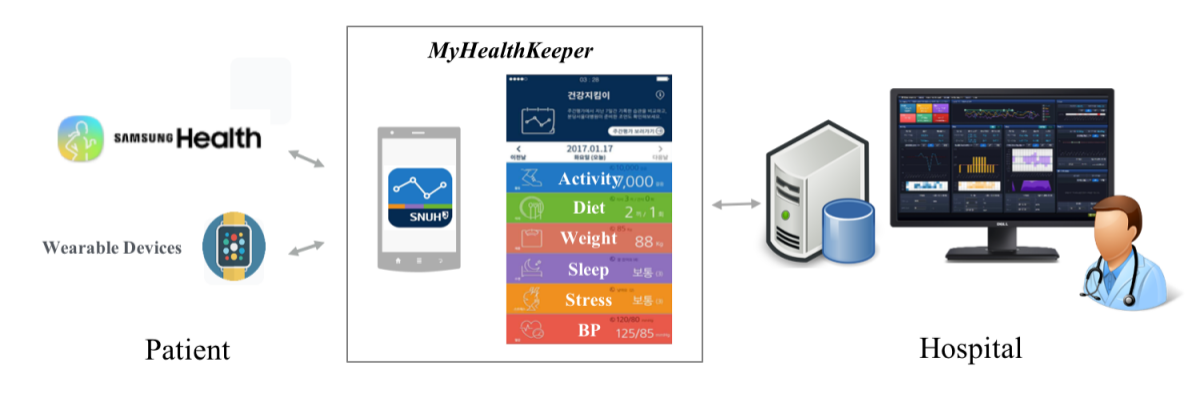
MyHealthKeeper personal health record development.

Our new PHR app, *MyhealthKeeper,* was developed to be compatible with the Samsung Health platform, which is one of the biggest worldwide mobile platforms, and to be linked to our hospital EHR system, Bestcare (ezCaretech Co, Seoul, South Korea; Figure 2).

**Figure 3:**
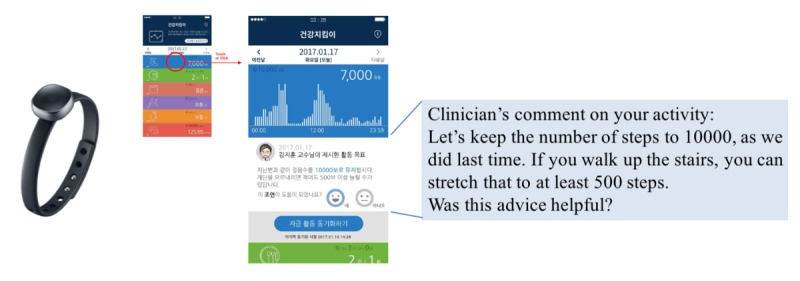
Clinician’s comment delivery on individual lifestyle.

The *MyHealthKeeper* interface was designed to work in the accordion format, which can be used to navigate through the tabs. The app was composed of several subpages for recording daily meal, sleep log, stress, blood pressure, weight value, and synced activity step counts. Weight value, subjective stress, sleep time, and sleep satisfaction logs were recorded once a day, whereas meal logs were recorded 3 times a day (breakfast, lunch, and dinner). Each meal record includes the input time and the amount on a 5-point scale. Daily step counts were collected through Samsung Health platform. Clinician feedback comments were implemented on each page to improve communication between doctors and patients (Figure 3). The patient’s response to the comments as well as other subpage usage logs were collected and analyzed for each group.

### General Characteristics of Clinical Trial Participants

Table 1 shows general characteristics of participants. A total of 60 patients (51 males and 9 females) were enrolled in this study. In addition, 43 patients (43 males) finished the study, whereas 17 patients (8 males and 9 females) were excluded because of withdrawal or incomplete follow-up sleep study. The analysis used a per-protocol methodology for the withdrawn patients. The mean age in app plus wearable, app-only, and control groups was 45.3, 41.5, and 40.5, respectively (*P*=.45). The BMI was 29.1, 30.8 and 28.7 in the app plus wearable, app-only, and control groups, respectively (*P*=.15). There were also no significant differences in demographics and smoking and drinking behaviors among the 3 groups.

**Table 1:** General characteristics of study participants.

Characteristics	App + wearable (n=15)	App-only (n=15)	Control (n=13)	*P* value
Age (year), mean (SD)	45.3 (9.5)	41.5 (11.8)	40.5 (7.4)	.45
Weight (kg), mean (SD)	84.3 (5.6)	93.7 (22.3)	84.3 (12.1)	.13
Body mass index (kg/m^2^), mean (SD)	29.1 (2.8)	30.8 (6.0)	28.7 (2.8)	.15
Height (cm), mean (SD)	170.4 (6.7)	174.0 (5.7)	171 (7.0)	.42
College or higher, n	13	12	13	.37
White collar, n	9	7	10	.36
Married, n	13	11	12	.44
Smokers, n	5	4	3	.77
Drink twice or more per week, n	5	6	6	.86

### Changes in Health Outcome: Analysis of the Primary and Secondary Outcomes

**Figure 4:**
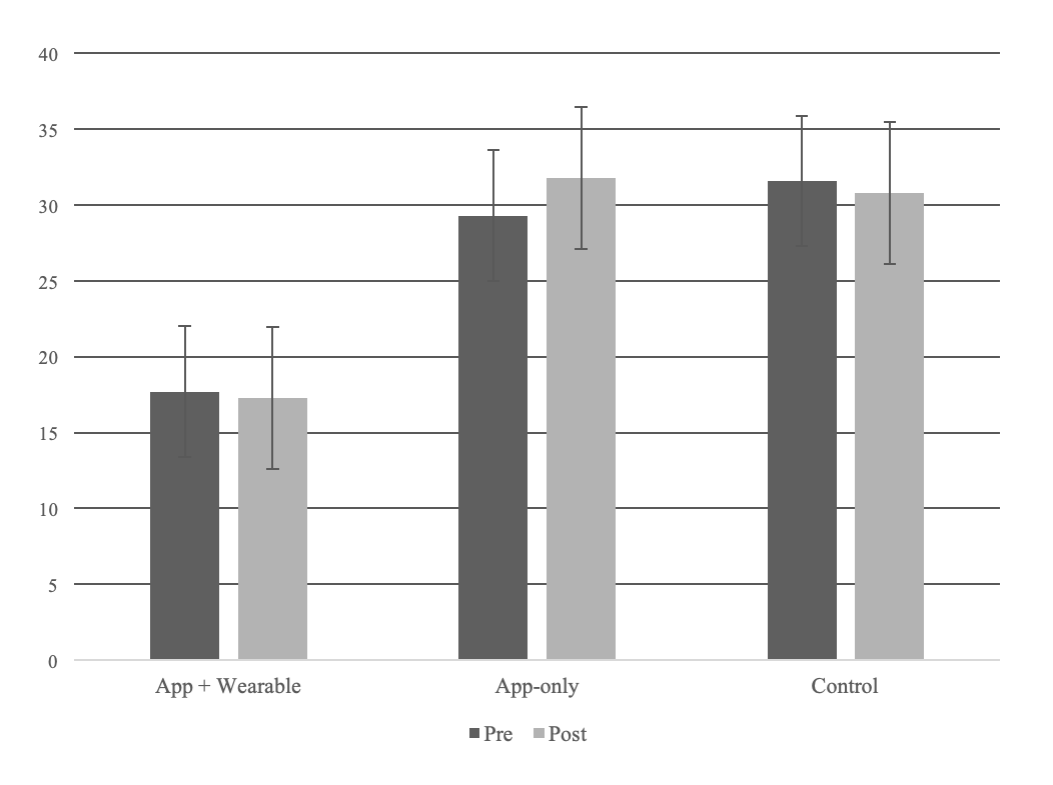
Pre- and postchanges in apnea-hypopnea index profile.

Our clinical trial study results revealed a significant change in weight loss in both intervention groups (both wearable device users and PHR app–only users). Moreover, 2 PHR intervention group participants who used the *MyHealthKeeper* mobile app every day showed significantly larger changes in weight and BMI than those in the control group (Table 2; average: 1.4 kg and 2 kg; 95% CI: 0.9-1.9; *P*<.001) In this study, there were no statistically significant changes in subjective improvement of respiratory system test results (Table 3 and Figure 4).

**Table 2:** Primary outcome changes of each group.

Characteristics	App + wearable (n=15)			App-only (n=15)			Control (n=13)		
	Pre, mean (SD)	Post, mean (SD)	*P* value	Pre, mean (SD)	Post, mean (SD)	*P* value	Pre, mean (SD)	Post, mean (SD)	*P* value
Weight (kg)	84.3 (5.6)	82.9 (5.6)	0.02	93.7 (22.3)	91.7 (22.9)	0.003	84.3 (12.1)	83.9 (12.6)	0.3
Body mass index (kg/m^2^)	29.1 (2.8)	28.7 (3.0)	0.02	30.8 (6.0)	30.1 (6.2)	0.002	28.7 (2.8)	28.6 (3.0)	0.34

**Table 3:** Subjective improvement of respiratory system.

Characteristics	App + wearable (n=15)				App-only (n=15)				Control (n=13)				*P* value^a^
	Pre, mean (SD)	Post, mean (SD)	*P* value^b^	Pre, mean (SD)		Post, mean (SD)	*P* value^b^	Pre, mean (SD)		Post, mean (SD)	*P* value^b^		
										
Snoring frequency (days/week)	5.9 (1.5)	4.7 (2.3)	0.11	5.4 (1.9)		4.2 (2.7)	0.04	6.2 (1.3)		4.3 (2.7)	0.02	0.79	
Apnea witnessed (days/week)	3.7 (2.7)	2.9 (2.8)	0.36	3.9 (2.6)		3 (2.7)	0.25	4.2 (2.7)		2.8 (2.7)	0.11	0.92	
Daytime sleepiness (days/week)	5.1 (1.9)	3.6 (2.2)	0.01	4.2 (2.4)		3.4 (2.4)	0.17	3.7 (2.7)		2.5 (2.4)	0.22	0.62	

^a^*P* value: pre- and postcomparison among the 3 groups*.*

^b^*P* value: pre- and postcomparison within each group.

### Patients’ Response: Comparison of Step Counts Between App Plus Wearable and App-Only Groups

**Figure 5:**
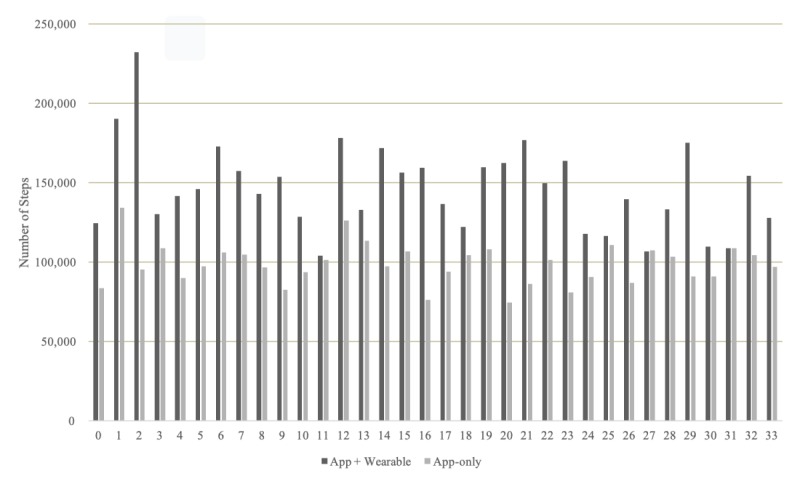
Changes in daily steps per group.

The average daily step counts (8165 steps) from the app plus wearable group were significantly higher than those (6034 steps) from the app-only group because they collected step-count data from different devices (*P*=.02). However, the diurnal hourly pattern of step counts was almost similar (Figure 5). Due to the nature of the collection devices, such as wrist wearable or mobile phone, the gait information was slightly different.

We assumed that each lifestyle data record is patient engagement reflection as patient response to PHR system and the clinician. The data collection record rate was calculated according to the records of 1 day. Study participants were moderately required to record daily weight, stress, snack, meal, and sleep logs. For daily diet records, which can be logged as breakfast, lunch, and dinner, if any of the 3 records were recorded, we processed as 1 record for that day.

The average rate of data collection was not different in physical activity (49.82% vs 49.96%; *P*=.99), food intake (32.67% vs 32.82%; *P*=.98), sleep (32.01% vs 32.45%; *P*=.95), stress (30.11% vs 27.33%; *P*=.70), and weight (32.82% vs 31.87%; *P*=.90) in the app plus wearable and app-only groups, respectively.

**Figure 6:**
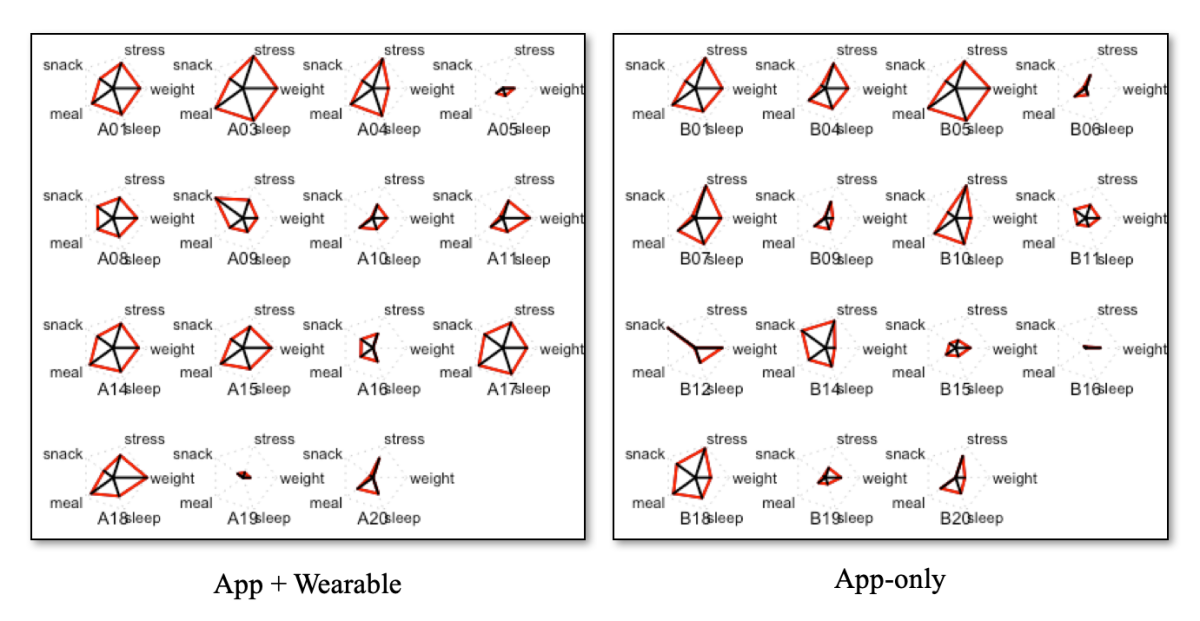
Star plot based on recorded lifelog per each group.

**Figure 7:**
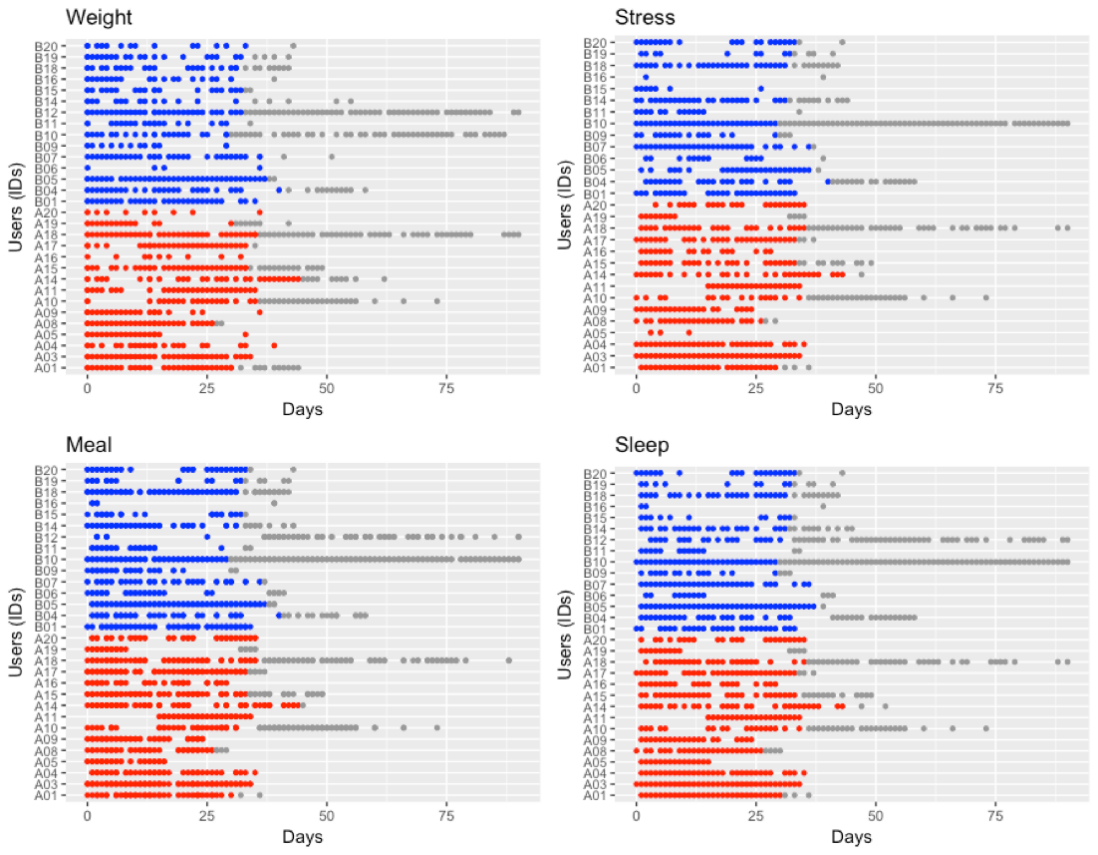
The number of recorded lifelog data for weight, stress, meal, and sleep logs (red: recorded days by app plus wearable users; blue: recorded days by app-only users; and gray: maintain period days without intervention).

We combined all the lifestyle data and data logging scores for each patient and derived a star-shaped pentagon plot with 5 types of lifestyle log data. The mean area value for each plot is a range of 0 to 1 by every user (Table 4). According to the composite lifestyle scores, we calculated the star-shaped pentagon plot area ranging from 0 to 1 (Table 4), and the plots were drawn as shown in Figure 6. Patients could be divided into 2 subgroups: compliant versus noncompliant, based on the average median value as 0.4 (Table 4), star plot square measure over 0.4 grouped as compliant. The percentage of compliant patients was 63.64% and 36.36% in the app plus wearable and app-only groups, respectively (*P*=.26). Figure 7 demonstrates lifelog records per each group: red color stands for recorded days by app plus wearable users, whereas blue color stands for recorded days by app-only users, and gray color is used to maintain period days without intervention.

**Table 4:** Star-shaped pentagon plot area calculation with meal, snack, stress, and sleep log records. The area value was normalized between 0 to 1. Each intervention group participant’s minimum, first quartile, median, mean, third quartile, and maximum value was described below.

Indices	Minimum	First quartile	Median	Mean	Third quartile	Maximum
App + wearable (n=15)	0.06	0.17	0.37	0.33	0.45	0.66
App-only (n=15)	0.01	0.09	0.14	0.25	0.4	0.67

### Patients’ Response: Usability Reflecting Patients’ Response to Automated Clinician Comments on Daily Lifestyle

**Figure 8:**
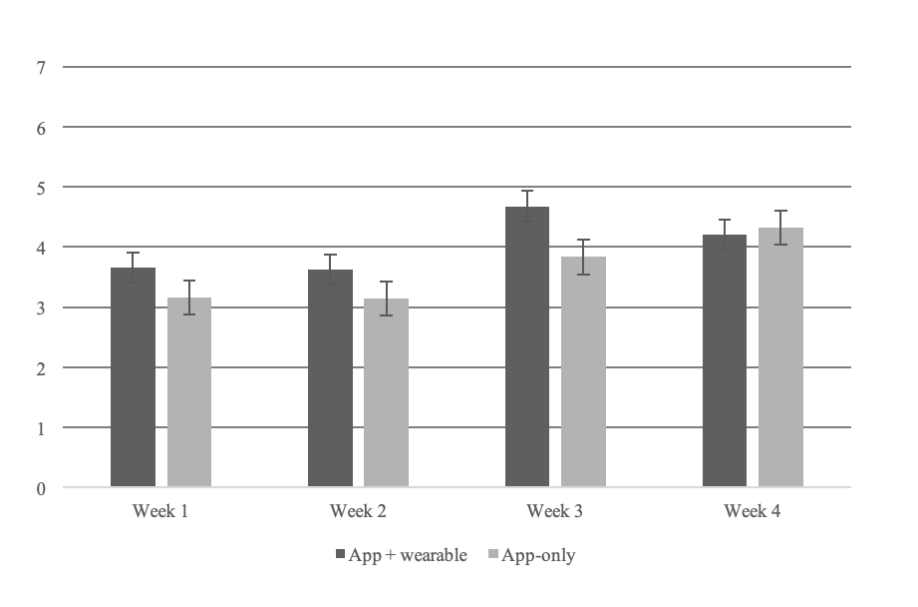
Patient response to clinician feedback comment.

To study health app usability based on patients’ responses to automated lifestyle comments, we analyzed usage logs for clinician comment feedback, which is depicted in Figure 3. Figure 8 demonstrates patients’ response to clinician feedback comment per each group as a satisfaction score. When a patient responded satisfied, the response was scored as 1, and 0 otherwise. App plus wearable users tend to answer more than app-only users in detail. Furthermore, these 2 intervention group participants showed increased answers between both weeks 2 and 3. At this point, patients visited to see their clinician and anthropometric measurement was done in the hospital, these actions may influenced to motivate PHR usage.

## Discussion

### Principal Findings

The mobile phone–based PHR system in this study has a flexible and unique platform architecture to collect patient-generated health data and deliver patient response to EHR for the following reasons. First, the user can use PHR service in this study only if he or she has a smartphone, regardless of whether he or she has a wearable device or not. It was available to record daily health logs with Android OS app, as one of the nation’s largest market share, and to connect the data with the most popular Samsung Health platform. To the best of our knowledge, this is the first study on the system that links EHR-tethered PHR with conventional health platform such as Samsung Health. Second, the 3-arm clinical trial design, which targeted to observe the impact of wearable device and mobile app, revealed that the reduction of weight was because of the use of our PHR system, not the use of the wearable. Given that the majority of mobile phones already have a health platform function such as activity tracking, using the mobile phone health platform itself might be enough for PHR health care service without wearables. We suggest that a long-term prospective study including a larger number of participants be warranted to show whether patients require a higher precision in step count to make weight loss decisions. Third, with EHR-tethered PHR system, clinicians are available to show their patient’s daily lifestyle and to deliver coaching feedback. Conventionally, clinicians just ask the patients to recall their lifelog or lifestyle to identify their lifestyles. Therefore, it seems to be quite inaccurate. The patient recall basis system is not enough for both patients and clinicians to pay much attention to the importance of lifestyle. However, the EHR-tethered PHR system may allow doctors and patients to check the patients’ lifelog information together on EHR screen and to prescribe lifestyle for patients. Therefore, clinicians can review these data on the PHR module interface on the EHR and provide health-related lifestyle management feedback to the patients during the patient’s visit to the clinic. From the experience using this system, patients are more likely to be interested in their lifelog data because the lifelog can be objectively summarized on the EHR and shared with their own clinicians.

Wearable biosensors are noninvasive devices used to acquire, transmit, process, store, and retrieve health-related data [26]. Biosensors have been integrated into various platforms, including watches, wristbands, skin patches, shoes, belts, textiles, and smartphones. Patients have the option to share data obtained by biosensors with their providers or social networks to support clinical treatment decisions and disease self-management [27]. In our previous study, we aimed to demonstrate the development of an EHR-tethered PHR app called *MyHealthKeeper*, which can retrieve data from a wearable device and deliver them to a hospital EHR system, and to study the effectiveness of PHR data–driven clinical intervention with clinical trial results [17]. We gathered this patient-generated lifestyle-related health information with a mobile app and activity tracking device and transferred it to a PHR data server to create a summary view based on the practical needs of the clinicians. Our first clinical study was a preliminary study aimed at observing the weight loss impact for obesity patients of conventional care versus EHR-integrated PHR–based care after system development.

The related previous study examined the patient usage logs with the 5-year use of a mobile PHR system distributed by a tertiary hospital in South Korea [15]. On the basis of actual mobile PHR usage data, they investigated the usage pattern and characteristics of the users of patient-generated health data services. This was the first approach to analyze long-term usage data in mobile PHR research [15]. In addition to user-specific characteristics (eg, age, sex, and diagnosis), the study suggested that more research should be conducted on the modifiable factors that affect use duration, to facilitate activities that promote continued use.

In this study, we aimed to observe the effects of a PHR–based health care app with 3 arms: the mobile app and wearables, mobile app alone, and control in patients with sleep apnea. We also aimed at examining the impact of the use of PHR and wearables on health outcome improvement and sustained use of the health app that can be associated with patient engagement. As a primary outcome, we observed OSA patient’s weight loss, BMI, and other sleep-related parameters to examine health outcome changes. Moreover, 2 intervention group patients showed weight loss and BMI change during the trial period. We also collected and analyzed lifelogs, which are dependent on health behaviors influenced by patient engagement (meal habits, weight control, diet, and exercise). Patient response was analyzed by mobile app records. To analyze usability and patient response, we assumed patient engagement by proxy through feedback logs from automated clinician comments on each lifestyle, which reflects patient activation.

This study is a unique approach rather than other studies based on the following characteristics. First, we collected 2 types of intervention group users’ (app + wearable vs app-only) actual usage data to investigate the difference. Although we did not find a statistical difference in sleep-related health outcome between the groups, this study design is thoroughly concerned with lifestyle-related disease and can be further examined as a long-term observation. Second, our analysis found the continuous usage pattern, including without intervention period. This intervention-free observation reflects the actual user pattern in mobile PHR app without consciousness.

### Limitations

This study could not provide a longitudinal observation of the EHR-tethered PHR system because of the practical constraints. Due to the short clinical trial period and the small number of study participants, it was difficult to determine a causal relationship, and the study did not provide information about the precise improvement in the health outcomes of PHR users. Nevertheless, we tried to observe the effect of PHR system for OSA patients, which is closely related to personal lifestyle-related sleep factor. Furthermore, with this integrated PHR system, we also expect longitudinal follow-up and continuous patient engagement in future studies. We hope to derive and apply many PHR features of an EHR-tethered PHR system for a variety of lifestyle-related disease management in further studies based on this study protocol.

## Appendix

Multimedia Appendix 1 Supplementary image describes MyHealthKeeper mobile interfaces.

Multimedia Appendix 2 CONSORT-EHEALTH checklist (V 1.6.2).

## Abbreviations

BMI: body mass index
EHR: electronic health record
mHealth: mobile health
OSA: obstructive sleep apnea
PHR: personal health record
SNUBH: Seoul National University Bundang Hospital
