# Supplementary figures and images for: Impact of Personal Health Records and Wearables on Health Outcomes and Patient Response: Three-Arm Randomized Controlled Trial
Source: JMIR Mhealth Uhealth. 2019 Jan 4;7(1):e12070. doi: 10.2196/12070 (PMC6682299; doi:10.2196/12070)

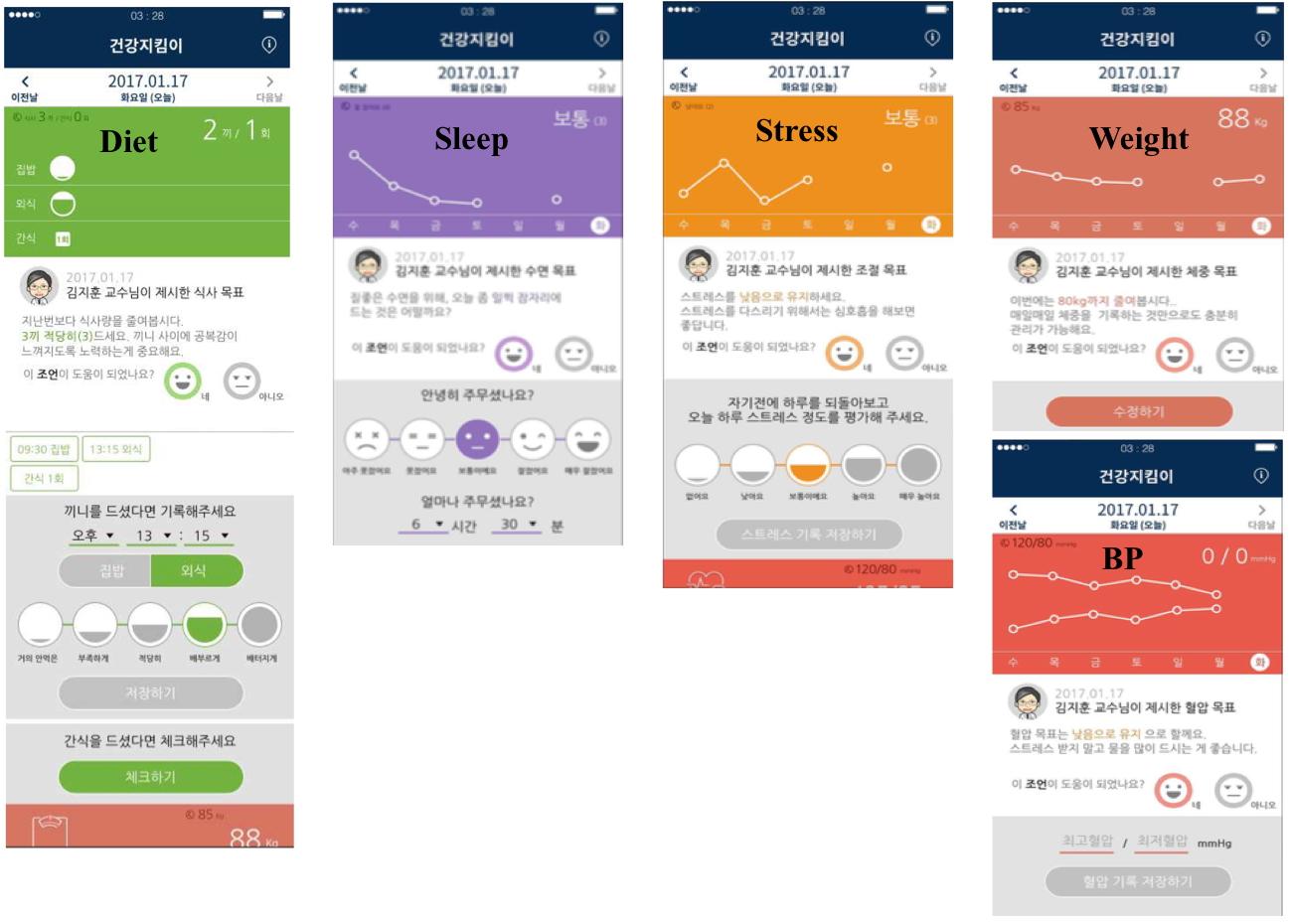

Supplement: Multimedia Appendix 1 [file mhealth_v7i1e12070_app1.png]
